# The Expression of CD74-Regulated Inflammatory Markers in Stage IV Melanoma: Risk of CNS Metastasis and Patient Survival

**DOI:** 10.3390/cancers12123754

**Published:** 2020-12-14

**Authors:** Dai Ogata, Jason Roszik, Junna Oba, Sun-Hee Kim, Roland L. Bassett, Lauren E. Haydu, Keiji Tanese, Elizabeth A. Grimm, Suhendan Ekmekcioglu

**Affiliations:** 1 Department of Melanoma Medical Oncology, The University of Texas MD Anderson Cancer Center, Houston, TX 77030, USA; dogata@ncc.go.jp (D.O.); jroszik@mdanderson.org (J.R.); joba@keio.jp (J.O.); sunhee.kim@mdanderson.org (S.-H.K.); egrimm@mdanderson.org (E.A.G.); 2 Department of Dermatologic Oncology, National Cancer Center Hospital, Tokyo 104-0045, Japan; 3 Department of Genomic Medicine, The University of Texas MD Anderson Cancer Center, Houston, TX 77030, USA; 4 Genomics Unit, Keio Cancer Center, School of Medicine, Keio University, Tokyo 106-8582, Japan; 5 Department of Biostatistics, The University of Texas MD Anderson Cancer Center, Houston, TX 77030, USA; rlbasset@mdanderson.org; 6 Department of Surgical Oncology, The University of Texas MD Anderson Cancer Center, Houston, TX 77030, USA; LEHaydu@mdanderson.org; 7 Department of Dermatology, Keio University, Tokyo 160-8582, Japan; tanese@a3.keio.jp

**Keywords:** melanoma, stage IV, prognosis, CNS metastasis, CD74, nitrotyrosine

## Abstract

**Simple Summary:**

Although many immunotherapies produce positive initial clinical responses, most advanced cancer patients recur so that there is an urgent need to identify and counteract both the intrinsic resistance as well as acquired mechanisms. During our studies on the mechanisms of resistance, we have identified a set of related protein markers, which we now employ to generate a useful signature of, associated with microenvironmental oxidative stress. Our study examines inflammatory marker expression in stage IV melanoma that are associated with survival outcome and risk of developing central nervous system (CNS) metastasis. Our data here presents CD74 as a prognostic tumor marker associated with good survival in stage IV melanoma. Additionally, the tumor cell nitrotyrosine (NT) expression predicts a greater risk of developing CNS metastasis in those patients. Our understanding of complex cancer cell and their response in the chronic inflammation environment would help us develop better treatments for melanoma.

**Abstract:**

Innate inflammatory features have been found in melanoma tumors from patients at all stages, and molecular analysis has identified definitive inflammatory proteins expressed by tumors cells in patients who presents the worst prognosis. We have previously observed weakened outcomes in patients with constitutive expression of inducible nitric oxide synthase (iNOS), macrophage migration inhibitory factor (MIF) and improved outcomes with CD74 expression in stage III melanoma. In our current study, we tested our hypothesis on CD74-regulated inflammatory markers’ expression in stage IV melanoma tumors whether the signature is associated with survival outcome and/or risk of developing CNS metastasis. We retrospectively identified 315 patients with stage IV melanoma. In a tissue microarray (TMA), we examined the expression of cells with CD74, its receptor MIF, and downstream inflammatory markers iNOS, nitrotyrosine (NT), cyclooxygenase (COX)-2 and microsomal prostaglandin E synthase-1 (mPGES1). We analyzed the association of those inflammatory markers with overall survival time (OS) and time to CNS metastasis using Kaplan–Meier survival analyses. Our data validates CD74 as a useful prognostic tumor cell protein marker associated with favorable OS as in stage III melanomas, while the tumor NT expression strongly predicts an increased risk of developing CNS metastasis (*p* = 0.0008) in those patients.

## 1. Introduction

According to the Melanoma Staging Database, early stage melanoma has an excellent prognosis, with 10-year survival rates as high as 95%, but survival rates decline to 8–20% at 10 years [1,2] when it metastasizes to the distant organs. In addition, melanoma has a relatively high affinity to metastasize to the central nervous system (CNS) [3,4]. The cumulative incidence of CNS metastasis in stage IV melanoma has been reported to be 15.8% at 5 years [5]. Although stage IV melanoma survival outcomes will continue to improve due to better treatment modalities, such as targeted therapy and immunotherapy, long-term survival rates are still low [6,7,8]. Hence, the determination of the precise regulating pathways and the associated individual markers that leads to the development of distant melanoma metastases—especially CNS metastases—is urgently needed. Our better understanding of the pathogenesis of the disease by investigating the molecular determinants of the CNS metastasis will eventually assist our decision on rational therapeutic approach for patients.

Despite the fact that controlled inflammatory response is beneficial, it could become detrimental if dysregulated. Inflammatory responses differ greatly depending on the nature of the instigators. By broad description, the tumor microenvironment has a central role in aiding metastasis and brain metastases are not any exception. In fact, due to the unique microenvironment of the brain (cellular exceptionality by oligodendrocytes and astrocytes along with others, such as pericytes, ependymal cells and infiltrating immune cells) it greatly differs from other organs. As a result, brain metastases are one of the deadliest of tumor metastasis and represents most dismal prognosis in general.

It has been previously shown that intrinsic tumor cell inflammatory mediators are associated with poor prognosis and is associated with the progression of melanoma as we discussed in our earlier review [9] in detail. We have also previously reported compromised outcomes in patients with constitutive expression of inducible nitric oxide synthase (iNOS), macrophage migration inhibitory factor (MIF) and better outcomes with CD74 expression in stage III melanoma [9,10]. In our current study, we tested our hypothesis on CD74-regulated inflammatory markers’ expression, CD74 itself, MIF, iNOS, nitrotyrosine (NT), cyclooxygenase (COX)-2 and microsomal prostaglandin E synthase-1 (mPGES1) in stage IV melanoma tumors and found that some are associated with survival outcome and the risk of developing CNS metastasis.

## 2. Results

### 2.1. Study Population

Our study population in this TMA, of which 169 (54%) did not have CNS metastasis at the time of the last follow-up, included tissue sections from 315 patients with distant metastatic melanoma. It included 223 (70.8%) male and 92 (29.2%) female patients with a median age of 56 years (range: 13–84 years). Twenty six patients had a brain metastasis as the first distant metastasis and 289 did not. At the end of this study, 81 patients were still alive and 234 were dead (Table 1). Our patient demographics represents common distribution of known factors, including relatively older age male population. Although gender is not directly and strongly associated, aging correlates with increased chronic inflammation which may play a role in reduced survival of older patients as well as their immune response to metastatic progression. Our panel of inflammatory markers were selected based on their involvement in our previous study of stage III melanoma [10] on their involvement with cancer inflammation. This panel is not inclusive nor complete as its current stage but further confirming inflammation’s role in advance disease.

Table 2 summarizes the overall configuration of the melanoma TMA. As presented here tissues of resected metastasis were from the lung (*n* = 163), intestine/colon (*n* = 85), liver (*n* = 20), adrenal gland (*n* = 13), spleen (*n* = 12), gall bladder (*n* = 5) and other (*n* = 17). The most common site of melanoma metastasis was the lung, consistent with previous reports [11].

### 2.2. Overall Survival (OS)

**Figure 1:**
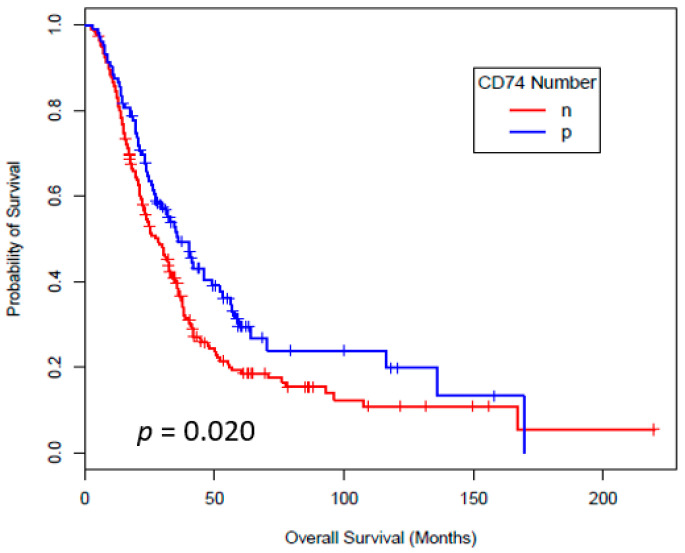
Overall Survival by CD74 Number. Clinical outcomes by CD74 in tumor cells in stage IV melanoma patients in datasets. Kaplan-Meier analysis for OS for the expression by percentage of staining. n = negative (<5% cells are positive), p = positive (=/>5% cells are positive).

Tissue sections from total of 293 patients were included in the OS analyses. Twenty two samples from the total of 315 patients were excluded based on the tissue sections quality to have a consensus scoring. Figure 1 shows the results of Kaplan-Meier survival analysis based on CD74 staining number. Tumor CD74 staining number (*p* = 0.020) and staining intensity (*p* = 0.020) both correlated significantly with a lower risk of death. As regards to other markers, there is no evidence of an association between those of expressions and OS in this cohort. The p-values for those markers were as follows; COX2 (*p* = 0.58), MIF (*p* = 0.29), mPGES1 (*p* = 0.19), iNOS (*p* = 0.99) and NT (*p* = 0.19), respectively (Figure S1).

**Figure 2:**
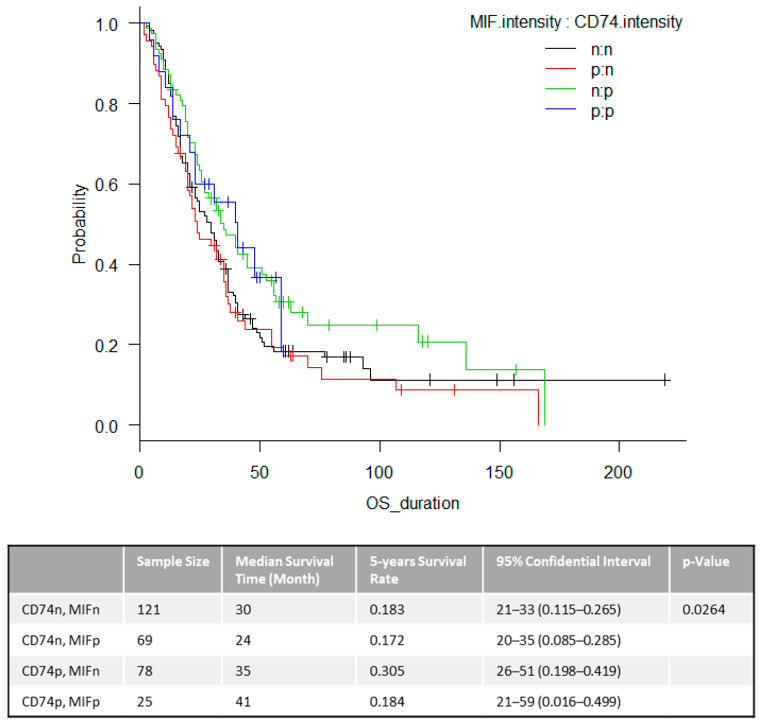
Overall survival for combined markers. Clinical outcomes by combined markers of CD74 and MIF in tumor cells in stage IV melanoma patients in datasets. Kaplan-Meier analysis for OS for their expression by intensity of staining. n = negative (no staining), p = positive (light to intense level of staining).

To further investigate this, in addition to the expression state of CD74, we assessed the combination of other markers for association with OS. As a result, the expression of CD74 and the loss of MIF only showed a favorable overall survival in stage IV melanoma patients (*p* = 0.0264) (Figure 2). This statistically significant relationship between OS and combined marker intensity provides an intriguing model for functional outcomes as this was also highly significant in our stage III study [10].

### 2.3. The Time to First CNS Metastasis

**Figure 3:**
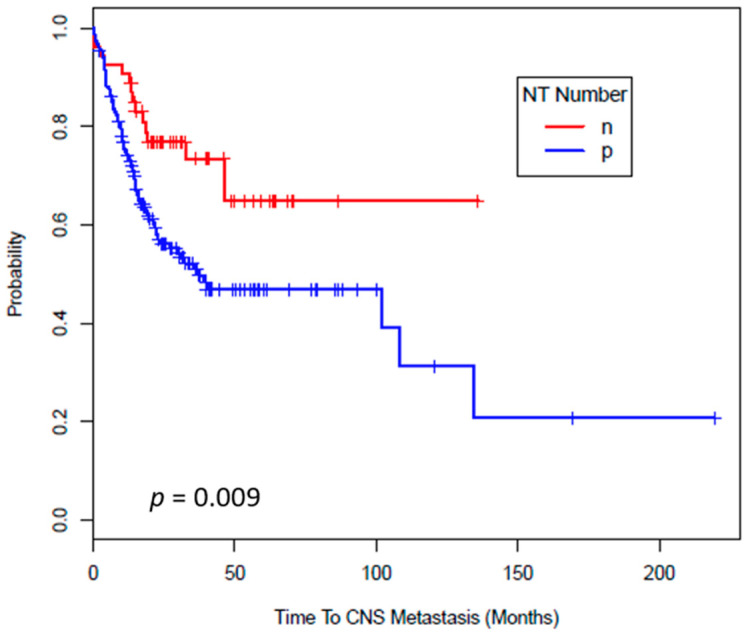
Time to CNS metastasis by NT Number. Kaplan-Meier survival estimates of the overall probability of developing CNS metastasis from the time of the stage IV diagnosis. In patients stratified based on the amount of NT expression, <5% versus ≥1%, in their systemic metastasis, there was significant difference in time to development of CNS metastasis (*p* = 0.009). n = negative (<5% cells are positive), p = positive (=/>5% cells are positive).

Figure 3 shows the results of Kaplan-Meier survival analysis based on number of positively stained tumor cells for NT. Tumor NT staining intensity (*p* < 0.01) and number (*p* = 0.009) both correlated significantly with time to CNS metastasis. The median time to development of CNS metastasis was 39 months (94% CI = 22–134 months) from the time of the stage IV diagnosis in patients whom tumor express NT versus it was not achieved (95% CI = 46-NA) in patients whose tumor do not express NT. In conclusion, patients, whose tumor express NT, experience CNS metastasis faster than patients who are NT negative. For all the other markers, CD74, COX2, MIF, mPGES1 and iNOS, we were unable to show any significant difference (Figure S2).

Figure 3 shows the results of Kaplan-Meier survival analysis based on number of positively stained tumor cells for NT. Tumor NT staining intensity (*p* < 0.01) and number (*p* = 0.009) both correlated significantly with time to CNS metastasis. The median time to development of CNS metastasis was 39 months (94% CI = 22–134 months) from the time of the stage IV diagnosis in patients whom tumor express NT versus it was not achieved (95% CI = 46-NA) in patients whose tumor do not express NT. In conclusion, patients, whose tumor express NT, experience CNS metastasis faster than patients who are NT negative. For all the other markers, CD74, COX2, MIF, mPGES1 and iNOS, we were unable to show any significant difference (Figure S2)

### 2.4. The Survival Associations in TCGA Stage IV Melanoma Dataset

**Figure 4:**
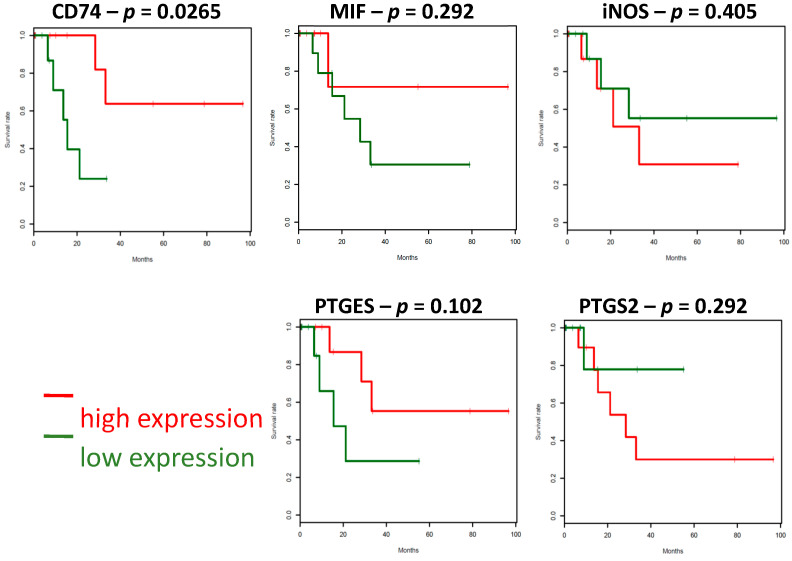
Survival associations in the melanoma TCGA dataset—Stage IV. Clinical outcomes by individual markers in tumor cells in stage IV melanoma patients in datasets. Kaplan-Meier analysis for OS for their expression of staining.

Recently, the cutaneous melanoma TCGA data set has become available (https://www.ncbi.nlm.nih.gov/pubmed/26091043), and we interrogated the expression of the markers of our interest. Our analysis was restricted to patients for whom the TCGA tumor biopsy was collected at the first diagnosis of stage IV disease (*n* = 77). Only high (above median) CD74 expression predicted better prognosis in stage IV melanoma patients when compared with low (below median) expression (*p* < 0.05) as shown below in Figure 4. This observation was unique to cutaneous melanoma as CD74 expression in uveal melanoma did not predict prognosis, rather represents trend for poor outcome (*p* = 0.16) Other inflammatory markers that involved in our protein panel, were also in agreement with our results that showing no statistical significance with MIF (*p* = 0.292), NOS2 (*p* = 0.405), PTGES (*p* = 0.102), PTGES2 (*p* = 0.292) (Figure 4 and Figure S1). Therefore, we present our findings here that we have identified (and validated by TCGA dataset) the protein expression of CD74 as providing survival information for stage IV melanoma prognosis.

### 2.5. Gene Expressions Associated with CD74 Expression in Stage IV Melanoma

To identify genes that are under- or over-expressed in samples with low or high CD74 expression in stage IV melanoma, we compared the expression of genes in the CD74 low (below median) and CD74 high (above median) sample groups.

**Figure 5:**
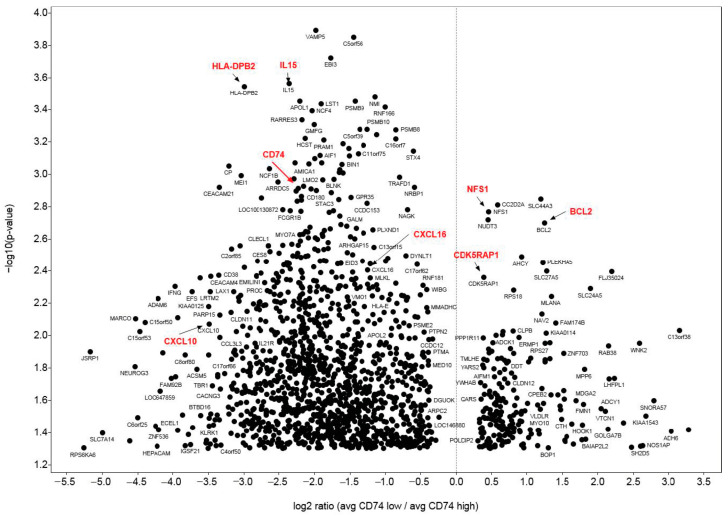
A Volcano Plot of the Genes in Association with CD74 in Stage IV Melanoma. The genes on the left side are lower in the “CD74 low” group compared to the “CD74 high” samples, while the genes on the right side have higher expression in the “CD74 low” samples.

The results are shown where the genes on the left side are significantly lower in the “CD74 low” group compared to the “CD74 high” samples, while the genes on the right side have significantly higher expression in the “CD74 low” samples (Figure 5). Overall, we observed immune-related genes, such as HLA Class II and IL15, or chemokines such as CXCL10 and 16 were significantly lower in the “CD74 low” group. However, cancer cell apoptosis genes such as CDK5RAP1 and BCL2 or iron-sulfur clusters, which protects cancer cells against oxygen to damage a class of iron-dependent proteins such as NSF1, are significantly higher in the “CD74 low” samples. These are individual pathways to be explored further and not part of our current study at this stage.

## 3. Discussion

Incidence of CNS metastases in cancer varies and melanoma has the highest risk of CNS metastasis among common solid tumors [12] and it is associated with a particularly poor prognosis since they may cause death in 60–70% of melanoma patients [13,14]. Despite the role of recent developments for the efficacy of immunotherapies in such a group of patients, identifying patients with CNS metastases in their early stages presents a strong rationale for predictive marker studies. However, identification of the predictive biomarkers for patient stratification has major limitations, perhaps until we efficiently use liquid biopsies of blood or CSF to design personalized treatment approaches after the primary tumors removal as well as monitoring the progression of the systemic disease.

Our analysis of two independent cohorts, which are one for protein in TMA and one for their genes in TCGA, implicated that increased CD74 expression is a marker of good prognosis in stage IV melanoma. These findings are consistent with our previously published study examining the positive impact of CD74 in stage III melanoma cohort [10]. Our results are also consistent with the previously demonstrated prognostic influence of CD74 on survival in patients with other types of malignancies, such as in basal-like subtype invasive breast cancer [15]. We moreover analyzed and found that CD74 expression correlated with higher levels of MHC class II expression by tumor cells and with a dense TIL response. CD74 is known primarily as the MHC class II invariant chain and functions in the molecular processing of MHC class II through the Golgi [16] and a key factor in anti-tumor immunity as an important component in the functional presentation of MHC class II restricted antigens [17,18,19]. Our understanding of complex cancer cell and immune response in the chronic inflammation environment would help us develop better treatments for melanoma. We currently envision that tumor CD74 functions in antigen presentation to the immune cells in the tumor microenvironment rather than acting as a receptor for an inflammatory protein MIF. However, the potential on developing various drugs targeting MIF’s binding to its receptor CD74 is still an attractive approach as it regulates most likely targets a part of the immune modulation [20,21,22]. If such an approach could regulate MIF’s immunosuppressing activity and pro-inflammatory arrangements, this would provide a therapeutic strategy if we only prevent CD74 function in antigen presentation to the immune cells in the tumor milieu. Their biologic partnership remains to be explored beyond their prognostic value, perhaps provides significant information for their value on predictive treatment decisions. In this current study, we have identified and validated the protein expression of CD74 (and together with MIF) as providing survival information, and propose that CD74+ and MIF− together be considered to form a “signature” for stage IV melanoma prognosis, as we found on stage III melanoma [10]. Moreover, in our initial efforts to understand CD74 role in melanoma progression, we performed similar analyses for CD74 on a melanoma progression TMA containing tissue cores from benign nevi, primary cutaneous melanomas, melanoma metastases to lymph nodes and visceral organs [23]. Our results showed that melanoma cells express CD74 associated with the progression from melanocytes and benign nevi to clinically evident melanoma but not any significant difference observed in earlier to late stages of disease. Overall, our findings provide potential clinical benefit by using CD74 in combination with active novel therapies in melanoma, including targeted therapies and/or immunotherapies.

In an opposite direction to these important findings, higher NT expression in melanoma tumors indicates poor survival and might contribute to tumor progression. Regulating the immune response by altering the microenvironmental oxidative stress could be an attractive strategy as it could activate the variety of transcription factors that lead to the differential expression of genes involved in inflammatory pathways. Modifying the NO functions could also be a further important tactic as it combines chemically with oxygen radicals to form ONOO− (peroxynitrite) to produce direct nitrosylation on numerous signaling proteins in the tumor cells. This reaction could be detected in these tumor tissues as a permanent product, called NT. Several groups showed that NT is an immune modulator that inhibits T lymphocyte activity and produces immune tolerance to tumor cells [24] and high expression of NT in antigen presenting cells inhibits their activity and reduces antigen-presenting efficacy. Nitration/nitrosylation reduces immunogenic peptides’ binding MHC molecules and could influence either T-cell receptor (TCR) contact or MHC class I and II contact positions, with significant harm on T cell responses [25,26,27]. Moreover, it could be toxic for lymphocytes and stimulate apoptotic cell death through protein tyrosine phosphorylation [28,29,30] which eventually leads to the lack of immune response. In tissue studies, it is found in thymic extracts and thymic sections and co-localized with apoptotic cells, suggesting that NT is also in thymic apoptosis [31]. Therefore, regulation of NT functions has great implication in regulating immune response to tumors. Our finding on the significant association between tumor NT expression and the time to CNS metastasis yet to be explored whether the lack of immune response causes their path to the CNS metastasis or tumor cells themselves regulates their path to metastasize into CNS.

Our study has limitations as our patient population was selected by retrospectively identifying patients from longer than a decade old samples with known outcome. More advanced treatment modalities have emerged during the last decade for stage IV disease, which clearly could not be included in this current study. Another limitation to our retrospective cohort study could have been loss to follow up which may cause significant validation concerns for our results. However, our patient selection included all individuals receiving any treatment during the study period, which may lead to smallest bias, if any.

Our data demonstrates CD74 as a useful prognostic tumor cell protein marker associated with favorable OS in stage IV melanoma as we have previously shown in stage III melanomas. Moreover, we have uniquely identified a significant association between tumor NT expression and the time to CNS metastasis and concluded that the tumor NT expression could predict an increased risk of developing CNS metastasis in those patients.

## 4. Materials and Methods

### 4.1. Melanoma Tissue Microarray (TMA)

Patients whose tumors were accessioned at MDACC between 1992 and 2010 included those with systemic, non-CNS metastasis samples, consisted of resected metastasis from the lung, intestine/colon, liver, adrenal gland, spleen, gall bladder and other non-CNS sites. Of these patients, 148 had or developed radiographic confirmed CNS metastases. The TMA was prepared from formalin-fixed paraffin-embedded (FFPE) tumors and contained two core samples from each specimen; tumors from patients with defined clinical outcomes and follow-up data.

### 4.2. Biomarker Assessments

**Figure 6:**
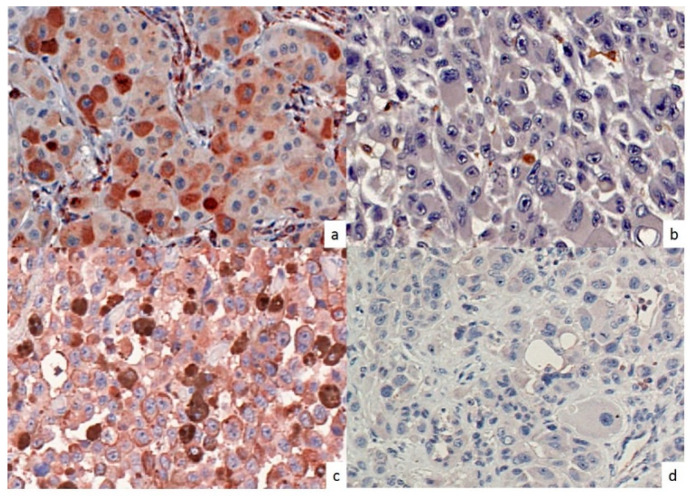
Tumor CD74 and NT expression characteristics by immunohistochemical analyses. Stage IV melanoma tissues from patients were immunostained for CD74 and NT. As published previously (10), both intensity and percentage of positive cells were quantified and protein expression associating with overall survival (OS) in this retrospective tissue microarray. Representative tumor CD74 positive (**a**) and CD74 negative (**b**), NT positive (**c**) and NT negative (**d**) specimens are shown (400× magnification). Both positive samples are scored as 3 for both intensity (intense staining) and number (>75% of cells stained) and negative samples are 0 for both intensity (no staining) and number (<5% of cells stained).

The protein markers assessed for this report were CD74, COX2, MIF, mPGES, and NO-driven post-translational modifications were also tested since antibodies to these have recently become available and are of much interest to our work; iNOS and nitrotyrosine (NT). We used anti-iNOS monoclonal antibody (Creative Biolabs, Shirley, NY, USA), a rabbit anti-NT polyclonal antibody (Millipore, Temecula, CA, USA), a rabbit anti-Cox-2 polyclonal antibody (Cell Signaling, Danvers, MA, USA), a mouse anti-CD74 monoclonal antibody (PIN.1, Novus Biologicals, Littleton, CO, USA), a goat anti-MIF polyclonal antibody and mPGES-1 (Novus Biologicals). Pre-immune normal mouse IgG (Vector Laboratories) and anti-vimentin antibody (Bio Genex Laboratories, San Ramon, CA, USA) were used as negative and positive controls, respectively. Antigen detection was performed using avidin-biotin-peroxidase complex [ABC] kit (Vectastatin, Vector Laboratories, Burlingame, CA, USA) and immunolabeling was developed with the chromogen 3-amino-9-ethylcarbazole. Immunolabeling was scored separately for two variables: (i) percentage of positively stained cells and (ii) intensity of staining of the positive cells as used by us previously (10). The complete absence of protein markers was defined as <5% of tumor cell expression when performed in parallel with positive expression in controls, such as endothelial cells in the same tissues. Thus, expression of a given protein marker was considered positive if the average positivity was at least 5% and evident intensity of the staining of which quantifications detailed below. Percentage scoring was considered positive if the average positivity was at least 5%. Intensity scoring staining was defined as follows: “0,” none; “1,” light; “2,” moderate; and “3,” intense, and reported as positive if an average score of at least 1 was obtained. Figure 6 shows representative IHC staining of CD74 and NT in a stage IV melanoma sample. All IHC analyses were independently interpreted by three readers (JO, KT, SE) without knowledge of the clinical data. Discrepancies in scoring were reconciled by a consensus reading between the readers.

### 4.3. TCGA and CCLE Data Analysis

Skin Cutaneous Melanoma data from The Cancer Genome Atlas (TCGA) (https://www.ncbi.nlm.nih.gov/pubmed/26091043) was obtained from public TCGA repositories. Melanoma cell line data was downloaded from the Cancer Cell Line Encyclopedia (CCLE; https://www.ncbi.nlm.nih.gov/pubmed/22460905).

### 4.4. Statistical Analysis

Two time-to-event outcomes, which were overall survival (OS), and the time to first CNS metastasis were calculated from the date of stage IV diagnosis. For OS, patients who remained alive were censored at the date of their last follow-up and the time to first CNS metastasis was computed from the date of stage IV diagnosis to the date of first CNS metastasis. Patients with earlier CNS metastasis or CNS metastasis at the time stage IV diagnosis were excluded (*n* = 26) from our analyses. Patients whom did not experience a CNS metastasis were censored by using the date of the patient’s last brain imaging assessment or the last date the patient was seen at MD Anderson Cancer Center.

All statistical analyses were executed using EZR software (Saitama Medical Centre, Jichi Medical University, Saitama, Japan), and a graphical user interface for R (The R Foundation for Statistical Computing, Vienna, Austria). OS and the time to first CNS metastasis were estimated using a Kaplan-Meier analysis. Univariate analysis (log-rank tests) and multivariate analysis (logistic regression analysis) were used to compare differences in event according to the expression of markers. A *p* value <0.05 was considered as statistical significance. This study was approved (LAB01-717 and LAB00-063) by the Institutional Review Boards of MD Anderson Cancer Center and complied with the 1983 revision of the Helsinki Declaration of 1975.

## 5. Conclusions

In conclusion, our data demonstrates CD74 as a useful prognostic tumor cell protein marker associated with favorable OS in stage IV melanoma as we have previously shown in stage III melanomas. In this study, we have uniquely identified a significant association between tumor NT expression and the time to CNS metastasis and concluded that the tumor NT expression could predict an increased risk of developing CNS metastasis in those patients. To the best of our knowledge, at this time no consistent reliable markers have yet to be validated for immune responsiveness. “Hot” or “inflamed T cell” suggests some immune considerations, but these adaptive T cell features at best appear complementary to our novel set of innate markers that are responsible for supporting an oxidative-stress environment. In this study we specifically aimed to clarify the predictive role and validate an initial set of immune-related and oxidative stress markers. The oxidative-stress induced immune response is known in classic immunology to protect “self” during the resolution of the oxidative destruction of foreign infectious agents. Higher levels of oxidants kill cells, but lower levels protect self from damage. Our discovery on the significant association between tumor NT expression and the time to CNS metastasis may reflect the absence of immune response and possibly causes the melanomas path to the CNS metastasis. As brain metastases are significant clinical problem in patients with melanoma, discovery of new markers and targeting of those significant ones by designing combinational approaches are immensely essential to overcome this dismal disease.

## Figures and Tables

**Table 1:** Patient demographics and survival status.

Patient Characteristics	*n* = 315
Gender, n (%)	
Male	223 (70.8%)
Female	92 (29.2%)
Age at stage IV (years), median(range)	56 (13–84)
Ulceration	
Yes	41
No	67
Missing	207
Breslow thickness (mm)	
N	131
Median (range)	2.05 (0.3–23.0)
Brain met at first distant met	
Exist	26
None	289
OS, *n* (%)	
Alive	81 (25.7%)
Dead	234 (74.3%)

**Table 2:** The distribution of examined organ tissues.

Tissue	Lung	Intestine	Liver	Adrenal	Spleen	Gall	Others	Total
N	163	85	20	13	12	5	17	315

## Supplementary Materials

The following are available online at https://www.mdpi.com/2072-6694/12/12/3754/s1, Figure S1: Overall Survival by Individual Markers, Figure S2: Time to CNS metastasis by Individual Markers.
