# Supplementary material for: The Expression of CD74-Regulated Inflammatory Markers in Stage IV Melanoma: Risk of CNS Metastasis and Patient Survival
Source: Cancers (Basel). 2020 Dec 14;12(12):3754. doi: 10.3390/cancers12123754 (PMC7764866; doi:10.3390/cancers12123754)
Supplement: Supplementary file 1 [file cancers-12-03754-s001.pdf]

cancers

MDPI

Supplementary Materials

The Expression of CD74-Regulated Inflammatory Markers in Stage IV Melanoma: Risk of CNS Metastasis and Patient Survival

Dai Ogata, Jason Roszik, Junna Oba, Sun-Hee Kim, Roland L. Bassett, Jr., Lauren E. Haydu, Keiji Tanese, Elizabeth A. Grimm and Suhendan Ekmekcioglu

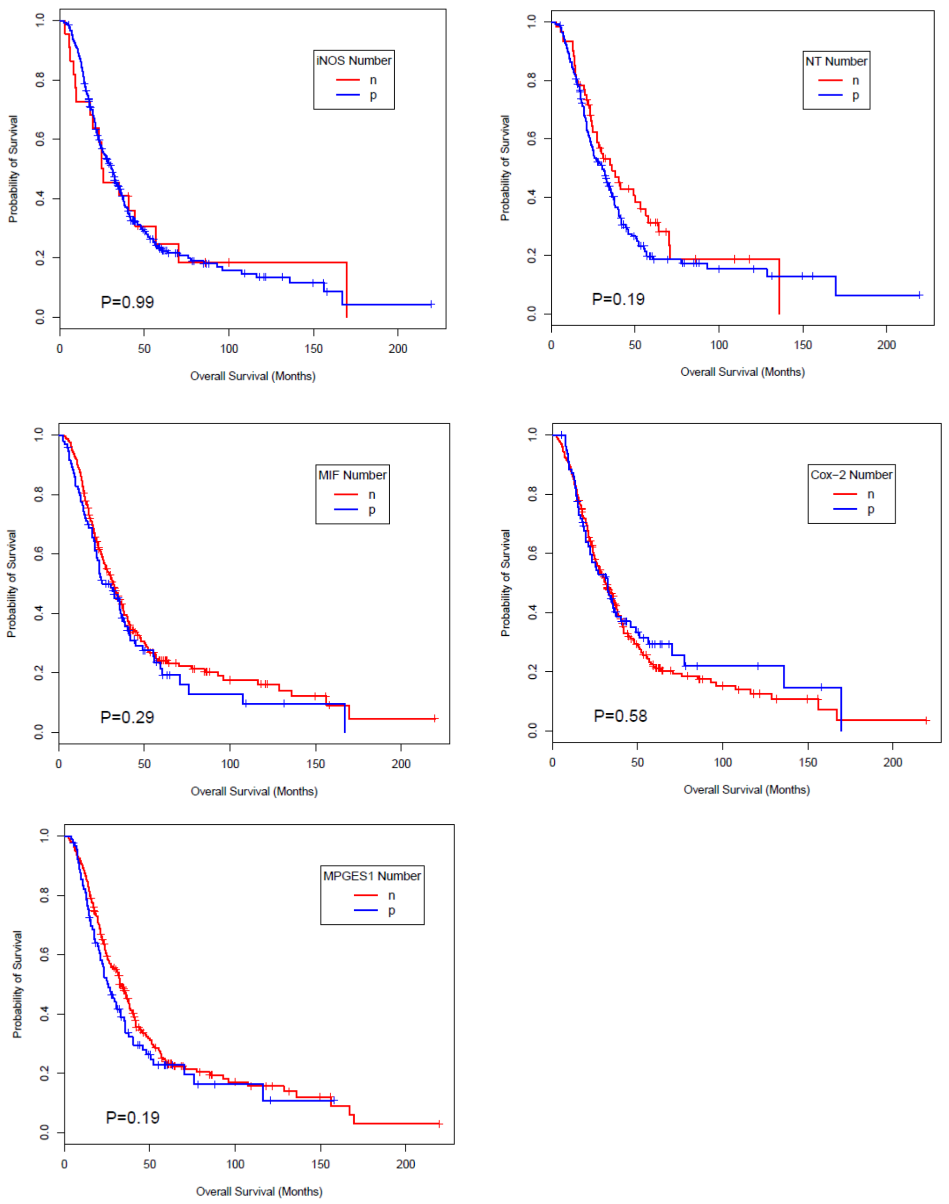

Figure S1. Overall Survival by Individual Markers.

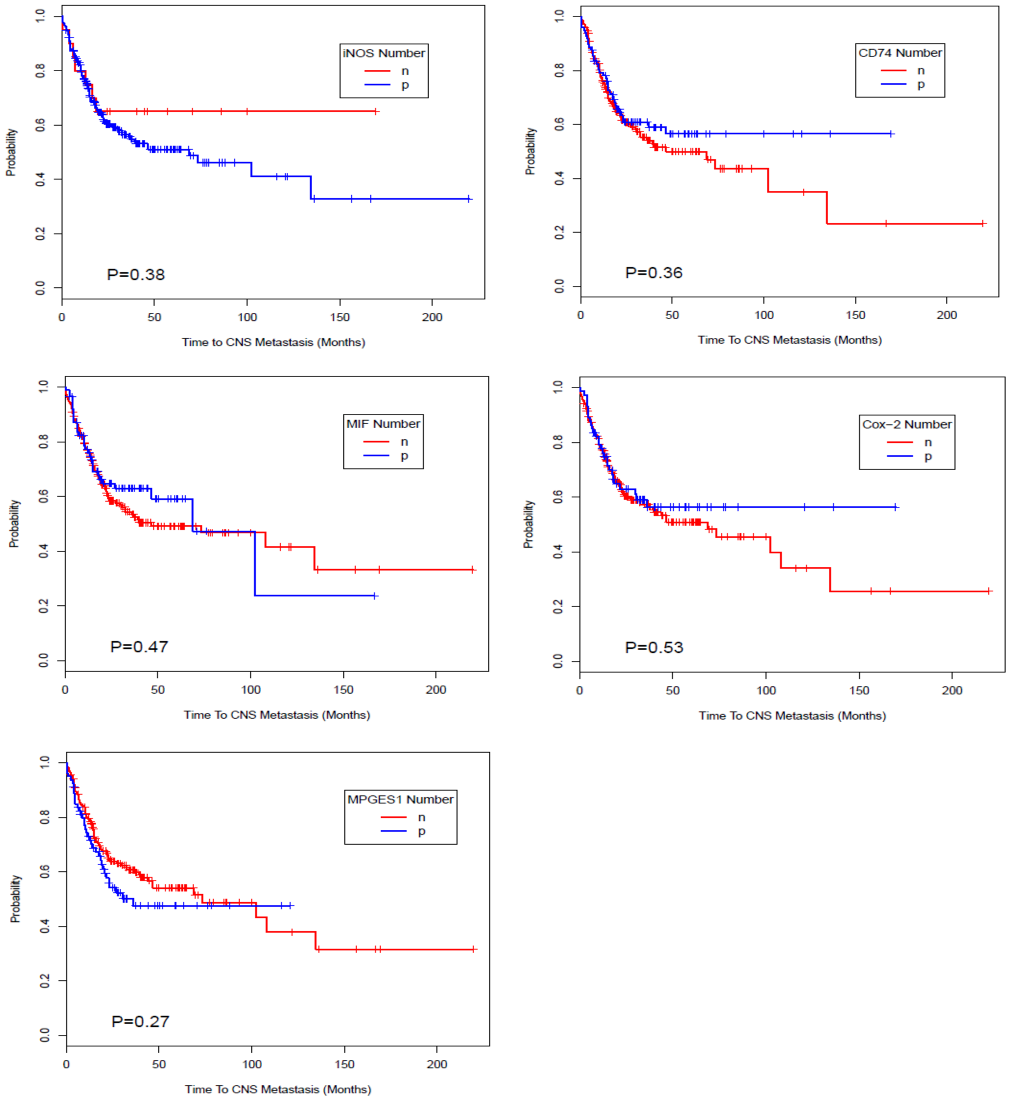

Figure S2. Time to CNS metastasis by Individual Markers.

© 2020 by the authors. Licensee MDPI, Basel, Switzerland. This article is an open access article distributed under the terms and conditions of the Creative Commons Attribution (CC BY) license (http://creativecommons.org/licenses/by/4.0/).
